# The role of imams and mosques in health promotion in Western societies—a systematic review protocol

**DOI:** 10.1186/s13643-016-0404-4

**Published:** 2017-02-02

**Authors:** Yassar Mustafa, Diya Baker, Preeti Puligari, Teresa Melody, Joyce Yeung, Fang Gao-Smith

**Affiliations:** 1 College of Medical and Dental Sciences, University of Birmingham, Edgbaston, Birmingham, B15 2TT UK; 2 Library, Birmingham Heartlands Hospital, Bordesley Green East, Birmingham, B9 5SS UK; 3 MIDRU, Birmingham Heartlands Hospital, Bordesley Green East, Birmingham, B9 5SS UK; 4 Institute of Inflammation and Ageing, College of Medical and Dental Sciences, University of Birmingham, Edgbaston, Birmingham, B15 2TT UK

**Keywords:** Health promotion, Imam, Mosque, Muslim, Narrative synthesis, Religious leader, Systematic review protocol, Western societies

## Abstract

**Background:**

Muslims comprise 4.8% of the national population in the UK and also form a significant proportion of its ethnic minority population, with trends set to continue for the foreseeable future. With ethnic minority health inequalities deepening further, there is an apparent lack of strategies to effectively tackle this growing problem. Imams, Muslim religious leaders, represent a hitherto under-investigated group who may have the capacity to facilitate positive health change within Muslim communities. The aim of this systematic review is to investigate the role of imams and mosques in health promotion in Muslim communities residing in Western societies.

**Method:**

We will undertake a systematic literature review of PubMed, CINAHL, EMBASE, MEDLINE, the Cochrane Library (CENTRAL) Register, NICE Evidence and Google Scholar. Eligible studies will primarily assess the role of imams and mosques in health promotion in Western societies. Secondary objectives include the identification of how mosque-based and imam-supported interventions were organised and delivered, and to explore which, if any, subgroups within the Western Muslim communities are more responsive to such interventions. Two independent reviewers will screen references from the electronic literature searches for eligible studies. The following data will be extracted to populate a tabulated form: study design, location of study, time of study, participant demographics, description of intervention, outcome measures of individual study, analysis methods, religious content (imams, mosques, religious denomination), outcomes and conclusions of study. Two investigators will independently assess the methodological quality of included studies. A narrative synthesis approach will be employed to analyse the extracted data in order to explore the role of imams and mosques in health promotion in Western settings.

**Discussion:**

This systematic review will elucidate the role and effectiveness of imams and mosques in health promotion in Western societies. If the use of imams and mosques is shown to be effective, this will encourage further research in Western Muslim communities that effectively utilise imams and mosques as part of novel strategies and interventions for health promotion in this group. The review will also aid policy makers in Western societies with a view to tackling and potentially reversing the problem of increasing ethnic minority health inequality.

**Systematic review registration:**

PROSPERO (CRD42015020166)

## Background

### Increasing Western Muslim population

Within the last few decades, Western societies have become increasingly ethnically diverse, with Muslims comprising a significant portion of the total ethnic minority population. For example, the UK Muslim population increased from approximately 50,000 in 1961 to approximately 2.7 million in 2011, forming 4.8% of the UK’s total population [1] and approximately 40% of its ethnic minority population [2]. Similarly, these population increases are paralleled in a number of other European countries such as Germany where there has been an increase from approximately 1000 in 1950 to 4.3 million Muslims in 2009 [3]. Moreover, with the global forecast predicting a 1.5% average annual growth rate of the global Muslim population, which is more than double the overall average growth rate of 0.7%, the current trends are set to continue in Western countries [4]. In the USA, for example, it is predicted that the Muslim population will rise from 1.349 million in 2010 to approximately 6.216 million in 2030 [5].

### Ethnic minority health inequalities

Consequently, ethnic minorities form an increasingly large proportion of the total number of patients treated in European and North American healthcare systems [6]. However, it is well established that there exists a large degree of health inequality amongst ethnic minority groups residing in Western countries, and which is currently increasing further [7]. The reasons for this health inequality and particularly the lack of engagement with health institutions are thought to be multifactorial and reasons include fear and mistrust of the health establishment within ethnic minority communities [8], language as a barrier to understanding [9] and perceived social stigmatisation [10]. Whilst the literature acknowledges these barriers, there is an apparent lack of strategies to effectively tackle and overcome these problems [11]. Indeed, a recent review of previously published evidence in the UK, Europe and USA concluded that a major contributory factor causing ethnic minority health inequality is ‘an extreme shortage of health promotion interventions’ [12].

### Use of imams (Muslim religious leaders)

One potential strategy that has been advocated is the use of religious leaders—trusted and well-respected figures in minority groups who may have the capacity to influence healthcare promotion and engagement on a communal scale [13, 14]. Ethnic minority religious leaders often wield a great deal of trust and thus influence over their respective communities, with some healthcare professionals now regarding their involvement in health promotion efforts as essential [15, 16].

Within the Muslim community, a religious leader is known as an ‘imam’ who primarily leads congregational prayers in the mosque setting. However, much like a parish priest, imams often take on additional roles within the Muslim community, including pastoral and advisory roles, conducting marriage ceremonies, teaching Islamic studies to children and arranging study circles for men and women. Indeed, they are often regarded as spiritual role models that can lead by example [17], play an important role in communal decision-making [18] and act as the gatekeepers for many social and cultural issues [19]. As such, imams may have the ability to positively facilitate local health promotion campaigns and thereby reduce existing health inequalities within this population. Therefore, a number of studies are calling for this potentially very effective method for health promotion to be further explored [20]. To date, however, the role of imams in promoting health in Western societies has not been systematically reviewed.

## Methodology

Reporting of this protocol will conform with the Preferred Reporting Items for Systematic Reviews and Meta-Analyses (PRISMA) guidelines [21]. This protocol is registered with PROSPERO (CRD42016042052).

### Research question

What is the role of imams (Muslim religious leaders) and mosques in health promotion in Muslim communities residing in Western societies?

### Objectives

The primary objective of the systematic review is to assess the effectiveness of imams (Muslim religious leaders) and mosques in health promotion in Muslim communities residing in Western societies.

Secondary objectives are as follows:

- To identify how mosque-based and imam-supported interventions were organised and delivered.
- To explore if some subgroups are more responsive to such interventions (e.g. based on ethnicity, religious denomination, gender or age).

Given the heterogeneity of study designs to be incorporated in this systematic review, responsiveness to any trialled interventions and their apparent effectiveness will be individually assessed taking into account any reported change in quantitative parameters as well as analysing any reported qualitative conclusions, either immediately post-intervention or over a longer time period, as relevant.

### Search strategy

The search strategy for this review is as follows:

1. Articles indexed in the following online bibliographic databases will be comprehensively searched: PubMed, CINAHL, EMBASE, MEDLINE and the Cochrane Library (CENTRAL) Register. Reference lists from retrieved papers will be searched and a grey literature search will also be undertaken using NICE Evidence and Google Scholar. The following search terms and their corresponding synonyms based on the research question will be used to locate relevant articles: ‘clergy’, ‘Islam’, ‘Muslim’, ‘Moslem’, ‘health promotion’, ‘healthcare delivery’ and ‘delivery of healthcare’.
  Relevant studies published in English will be retrieved with no time limits applied. This will ensure that trends will be monitored in the past few decades and will help to provide a more contextualised synthesis of results, including how the role of imams in health promotion in Western societies may be in a state of flux, potentially increasing or decreasing due to certain factors, in specific periods and sub-groups.
  Results from ‘Western societies’ will be analysed rather than the Muslim world itself, as per the research question, in order to focus on the effectiveness of religious leaders in a minority group setting, where the dynamics of their influence would differ significantly from a non-minority group setting. A sample search strategy from MEDLINE is displayed in Table 1.
2. Two reviewers (YM, DB) will independently screen the titles and abstracts of all the articles contained within the search results to assess their potential eligibility.
3. The full manuscripts of articles deemed potentially eligible will be retrieved, stored and managed for further investigation by two reviewers (YM, DB), using Mendeley, a reference management tool. Those papers that do not meet the eligibility criteria detailed below will be excluded.
4. The reference lists of eligible articles will be additionally searched for further relevant papers.
5. The full articles of all studies meeting the eligibility criteria will be reviewed and analysed.

**Table 1:** Sample search strategy for MEDLINE

	Search terms
1	exp CLERGY/
2	imams.ti,ab
3	mosques.ti,ab
4	exp ISLAM/
5	exp DELIVERY OF HEALTH CARE/
6	1 OR 2
7	3 OR 4
8	5 AND 6 AND 7
9	6 OR 7
10	5 AND 9
11	10 [Limit to: (Language English) and Humans]

#### Inclusion criteria

- The study sampled Muslims or ethnic groups that have a pre-dominantly Muslim religious affiliation.
- The study was undertaken in Europe (any country) or North America, Australia, New Zealand or South Africa.
- The study evaluated interventions targeted at any aspect of health promotion in the mosque setting or by using imams (Muslim religious leaders).
- The study reported relevant empirical findings.
- All study designs including experimental, observational, qualitative, evaluative and theoretical will be considered.

#### Exclusion criteria

- Multi-religious or multi-ethnic studies which do not report the percentages of specific ethnic or religious groups
- Studies where the religious community of interest constitutes less than 50% of the total study population—to minimise the effect of confounders (this strategy has been used in other systematic reviews of this nature (http://www.ncbi.nlm.nih.gov/pubmedhealth/PMH0051522))
- Studies undertaken in a Muslim-majority country—to focus on the role of imams in health promotion in a minority-setting where the intra-societal dynamics will be significantly different from within the Muslim world
- Studies written in a language other than English—for pragmatic reasons

#### Data extraction

From each study included in the systematic review, the following data points will be extracted and inputted into tabulated form onto a pre-designed pro-forma:

- Study design—as all study designs will be included in this systematic review, this will be an important differentiator.
- Location of study—by including city and country, this will allow investigation as to any similarities or differences between various Western Muslim communities.
- Year of study—this will help to reveal any trends in the imam’s role in health promotion particularly due to concurrent factors and may give more insight into the responsiveness to the intervention and its effectiveness.
- Participant demographics—including number, age, gender and ethnicity.
- Description of intervention—if applicable, control and intervention populations, type of intervention, eligibility criteria, sampling and randomisation.
- Outcome measures—these will vary depending on the individual study design.
- Analysis methods—a description of the quantitative or qualitative methods that were employed, if applicable.
- Religious content—the extent of involvement of mosques and imams, the qualifications of religious leaders, and the religious denomination(s) which were included.
- Outcomes—relevant outcomes from individual studies demonstrating an effect on health engagement or promotion will be reported.
- Conclusions—relevant conclusions from included studies to investigate the primary and secondary objectives of the systematic review.

### Assessment of risk bias and methodological quality

Two investigators will independently assess the methodological quality of included studies. The Critical Appraisal Skills Programme (CASP) checklist for reviews will be used to assess the quality of adapted systematic reviews, the quality appraisal tool developed by the Effective Public Health Practice Project (EPHPP) to assess intervention and observational studies, and the CASP checklist for qualitative research to assess qualitative studies. In the event of any disagreement regarding the selection of a manuscript or data extraction, resolution will be achieved either by mutual consensus or, alternatively, arbitration by a third reviewer (JY).

### Data synthesis

Given the heterogeneous nature and anticipated diversity of outcomes, we will conduct analysis of the data by using a narrative synthesis approach described by Popay et al. [4] to explore the role of imams and mosques in health promotion. The four key components of the framework we will use include the following:

1. Developing a theory—on the role and effectiveness of imams and mosques in supporting health promotion programmes and efforts; and if appropriate, exploring how and why these interventions work.
2. Developing a preliminary synthesis—given the heterogeneity in study designs, tabulation of the included studies informed by the research objectives will transform the data into a format conducive for accurate textual descriptions and analysis.
3. Exploring relationships in the data—by comparing and contrasting similarities and differences between conclusions, recurring cross-sectional themes highlighted in the included studies will be identified; with a minimum of three references to a theme from three independent papers constituting a cross-sectional theme.
4. Assessing the robustness of the synthesis—critical reflection will allow assessment of the limitations and generalisability of the narrative synthesis and highlight possible areas for future research.

## Discussion

By undertaking a systematic review of the literature, the role and effectiveness of imams and mosques in health promotion in Western societies will be elucidated. The systematic review findings will contribute to a deeper understanding of their role in a religious group that largely comprises ethnic minority populations in Western societies, and in whom health inequalities are not only very prevalent but deepening as compared to the national population. This review will therefore aid policy makers in Western societies with decision-making regarding tackling ethnic minority healthy inequality. It will also provide guidance for the focus of future research by identifying gaps in the current research literature and elucidate the scope for novel and impactful interventions using imams and mosques in this targeted population.

## Abbreviations

CASP: Critical Appraisal Skills Programme
CENTRAL: Cochrane Library hosts the Central Register of Controlled Trials
CINAHL: Cumulative Index of Nursing and Allied Health Literature
EMBASE: Excerpta Medica Database
MEDLINE: Medical Literature Analysis and Retrieval System
NICE: National Institute for Health and Care Excellence
NIHR: National Institute for Health Research
PRISMA: Preferred Reporting Items for Systematic Reviews and Meta-Analyses
